# Cophylogeny of the anther smut fungi and their caryophyllaceous hosts: Prevalence of host shifts and importance of delimiting parasite species for inferring cospeciation

**DOI:** 10.1186/1471-2148-8-100

**Published:** 2008-03-27

**Authors:** Guislaine Refrégier, Mickaël Le Gac, Florian Jabbour, Alex Widmer, Jacqui A Shykoff, Roxana Yockteng, Michael E Hood, Tatiana Giraud

**Affiliations:** 1 Ecologie, Systématique et Evolution, Bâtiment 360, Université Paris-Sud, F-91405 Orsay cedex, France ; CNRS F-91405 Orsay cedex, France; 2 Department of Zoology, 6270 University Boulevard, Vancouver BC V6T 1Z4, Canada; 3 MNHN UMR 5202, Unité Origine, structure et évolution de la biodiversité, Département Systématique et Evolution, 16 rue Buffon CP 39 75005, France; 4 ETH Zurich, Institute of Integrative Biology, Plant Ecological Genetics, Universitätstr. 16, 8092 Zurich, Switzerland; 5 Department of Biology, McGuire Life Sciences Building, Amherst College, Rts 9 & 116, Amherst, MA 01002-5000, USA

## Abstract

**Background:**

Using phylogenetic approaches, the expectation that parallel cladogenesis should occur between parasites and hosts has been validated in some studies, but most others provided evidence for frequent host shifts. Here we examine the evolutionary history of the association between *Microbotryum *fungi that cause anther smut disease and their Caryophyllaceous hosts. We investigated the congruence between host and parasite phylogenies, inferred cospeciation events and host shifts, and assessed whether geography or plant ecology could have facilitated the putative host shifts identified.

For cophylogeny analyses on microorganisms, parasite strains isolated from different host species are generally considered to represent independent evolutionary lineages, often without checking whether some strains actually belong to the same generalist species. Such an approach may mistake intraspecific nodes for speciation events and thus bias the results of cophylogeny analyses if generalist species are found on closely related hosts. A second aim of this study was therefore to evaluate the impact of species delimitation on the inferences of cospeciation.

**Results:**

We inferred a multiple gene phylogeny of anther smut strains from 21 host plants from several geographic origins, complementing a previous study on the delimitation of fungal species and their host specificities. We also inferred a multi-gene phylogeny of their host plants, and the two phylogenies were compared. A significant level of cospeciation was found when each host species was considered to harbour a specific parasite strain, *i.e. *when generalist parasite species were not recognized as such. This approach overestimated the frequency of cocladogenesis because individual parasite species capable of infecting multiple host species (*i.e. *generalists) were found on closely related hosts. When generalist parasite species were appropriately delimited and only a single representative of each species was retained, cospeciation events were not more frequent than expected under a random distribution, and many host shifts were inferred.

Current geographic distributions of host species seemed to be of little relevance for understanding the putative historical host shifts, because most fungal species had overlapping geographic ranges. We did detect some ecological similarities, including shared pollinators and habitat types, between host species that were diseased by closely related anther smut species. Overall, genetic similarity underlying the host-parasite interactions appeared to have the most important influence on specialization and host-shifts: generalist multi-host parasite species were found on closely related plant species, and related species in the *Microbotryum *phylogeny were associated with members of the same host clade.

**Conclusion:**

We showed here that *Microbotryum *species have evolved through frequent host shifts to moderately distant hosts, and we show further that accurate delimitation of parasite species is essential for interpreting cophylogeny studies.

## Background

Host-specific differentiation of parasites, also referred to as specialization, may arise as a consequence of limited dispersal or adaptive constraints [1]. Some parasite species or lineages may indeed have evolved a restricted host range simply because they have not come in contact with other host species, for instance when these occur in allopatry. Alternatively, host specificity may arise because of adaptive specialization [2], where trade-offs or fitness costs of being generalist parasites can lead to host-specific differentiation even in sympatry [1-4].

Host specificity is often expected to lead to cospeciation, *i.e. *parasite speciation tracking that of the host. For inferring whether cospeciation has occurred, one usually compares host and parasite phylogenies. Cospeciation yields congruent phylogenies (*i.e. *parallel cladogenesis), as has been previously illustrated, for example in the cases of animal hosts and parasitic lice [5] and of some mutualist associations [6,7]. In contrast, the colonization of new hosts, either followed by parasite speciation (host shift) or not ('failure to speciate' [8]), generally decreases phylogenetic congruence [9,10]. Additional processes are also expected to reduce the congruence between host and parasite phylogenies, such as species extinctions [5] and duplications (*i.e. *speciation of the parasite in absence of host speciation).

Several recent studies comparing the phylogenies of highly specific parasites and their hosts revealed widespread incongruence [9,11-14], showing that apparently strict host specificity is not sufficient to impede host shifts over the long term. In cases of host shifts, colonization is expected to be most likely between geographically overlapping hosts. The probability of the parasite being able to develop on a new host may also be influenced by the degree to which the new host shares chemical, physiological and ecological characters with the original host. Investigating geographical and ecological similarities between hosts can therefore help reconstructing evolutionary history of host shifts.

In addition, if related parasites more easily infect hosts with similar ecologies and if chemical, physiological and ecological characters in part covary with phylogeny, false conclusions can arise from cophylogeny analyses. In the first place, host shifts will preferentially occur between phylogenetically closely related hosts [15], which can generate similar degrees of congruence between parasite and host phylogenies as would cospeciation [16]. Second, generalist parasites that can infect several host species are likely to be found on closely related host species, either because these generalist species are the result of a lack of parasite speciation following host speciation or of host range expansion among phylogenetically close hosts that share biochemical and ecological characteristics. If generalist species are not recognized as such, intraspecific nodes in parasite phylogenies may be mistaken for speciation events and then misinterpreted as cospeciation events if the generalist parasites infect sister host species. This latter potential pitfall in cophylogeny analyses has not been investigated yet to our knowledge.

The anther smut fungus *Microbotryum violaceum *(Pers.: Pers) Deml & Oberw. (= *Ustilago violacea *(Pers.) Fuckel) (Basidiomycota: Pucciniomycotina, Microbotryaceae) is an obligate parasite on many Caryophyllaceae. It has been recorded on 92 plant species in Europe and on 21 plant species in North America [17]. The Caryophyllaceae – *Microbotryum *pathosystem is a model in many fields of evolutionary biology [18-20]. In diseased plants, diploid teliospores of *M. violaceum *replace pollen in the anthers, and are dispersed by insect visitors. Host specific divergence of *M. violaceum *has been of debate for about a century. Spore color [21], mating behavior [21], morphological differences [22], and cross-inoculation experiments [23,24], all suggested that *M. violaceum *strains found on different host species were at least partially differentiated. More recently, genetic analyses of *M. violaceum *populations from many hosts have revealed strong genetic differentiation [25-28]. Finally, an approach using multiple gene phylogenies has firmly established that most host races of *M. violaceum *represent multiple independent evolutionary lineages, highly specialized on a single or a few host species [29]. *Microbotryum violaceum *is thus a complex of more than fifteen true sibling species, showing a strong post-zygotic isolation [30].

The host family, Caryophyllaceae, has a global distribution with highest diversity in the holarctic, but also high diversity in the Mediterranean and Irano-Turanean region [31]. The majority of the approximately 2,200 species of the family are heliophytes occurring in dry, open habitats. Some species are restricted to mountainous regions and the family is totally absent from lowland rain forests [31]. *Microbotryum violaceum *commonly causes disease on species from two subfamilies of the Caryophyllaceae, the Alsinoideae and the Caryophylloideae, and is most prevalent on perennials [17]. The systematics of the Caryophyllaceae still mainly relies on morphological characters although there have been recent efforts to reconstruct the phylogenetic relationship among genera based on molecular data [32-34]. This is unfortunately insufficient for addressing questions on the Caryophyllaceae – *Microbotryum *association.

The goals of this paper are to reconstruct the evolutionary history of associations between the *Microbotryum *anther smut fungi and their Caryophyllaceae hosts and to test the effect of parasite species delimitation on cophylogeny analyses. We addressed the following questions:

1) What is the evidence for cospeciation and/or host shifts in this system?

2) Does delimitation of cryptic fungal species influence the results of cophylogeny analyses?

3) Do host shifts occur preferentially onto phylogenetically close hosts?

4) Does geography explain parasite similarity?

5) Do ecological factors, such as pollinator spectra or plant habitat, influence host shifts?

## Results

### Host phylogeny

**Figure 1:**
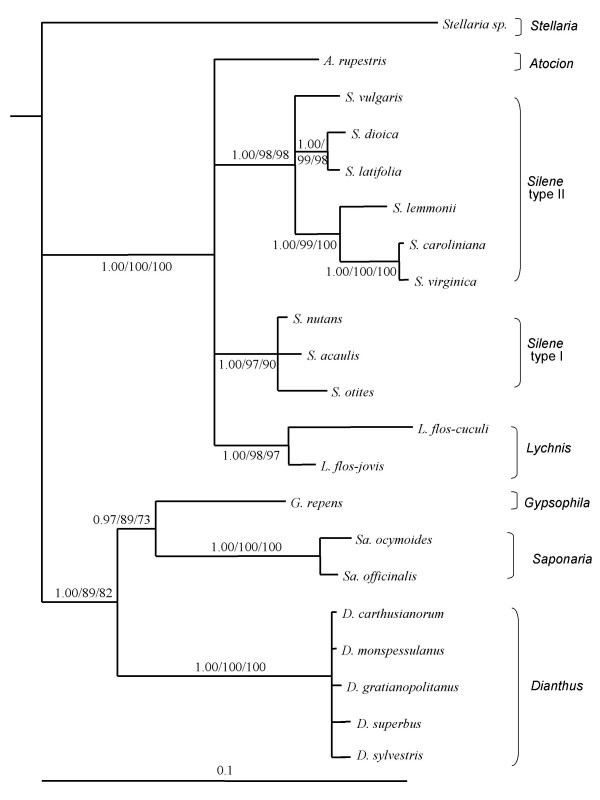
**Bayesian 50% majority-rule consensus tree of the Caryophyllaceae hosts used in this study based on the concatenation of the ITS, intron trnL and spacer trnLF**. Statistical supports indicate Bayesian Posterior Probabilities (BPP)/Maximum Parsimony Bootstraps/Neighbor-Joining Bootstraps. Only nodes supported by more than two methods are indicated, the significant statistical supports being considered as higher than respectively 0.9/70/70. The tree is rooted based on previous studies (see text).

The topologies of the host phylogenetic trees based on the ITS, intron *trn*L and spacer *trn*LF sequences showed no significant incongruence neither when assessed via an Approximately Unbiased (AU) tests (Table 1) nor when inspected by eye (see Methods for details). A tree was therefore constructed using the concatenated loci (Fig. 1). This tree adds support to the monophyly of the genera *Dianthus*, *Saponaria*, and *Lychnis*, as previously reported for a smaller set of species [33,35]. Within *Silene*, we identified two highly supported clades, that we named Silene type I and Silene type II.

**Table 1:** Results of the AU (Approximately Unbiased) tests for the plant dataset.

		Enforced topology (MP tree with bootstraps > 70)		
		
		ITS	trnL	trnLF
Gene used	ITS	0.612	0.326	NA
	trnL	0.388	0.630	NA
	trnLF	0.388	0.301	NA

*P*-values lower than 0.05 indicate that the likelihood of the topology obtained using one focal gene is significantly different from the likelihood of the enforced topologies (obtained using each other gene). NA indicates that no tests were possible because the phylogenetic relationships potentially incongruent between the alternative topologies were not resolved in the focal topology.

### Smut phylogenies and identification of phylogenetic species

**Figure 2:**
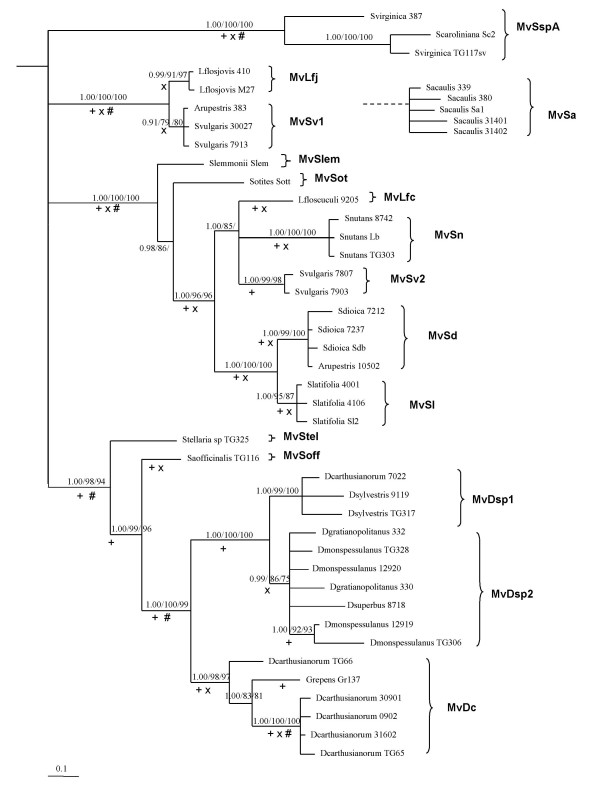
**Bayesian 50% majority-rule consensus tree of the *Microbotryum *strains analyzed in this study based on the concatenation of β-tub, γ-tub and EF1α sequences, and delimitation of the corresponding species**. Statistical supports indicate Bayesian Posterior Probabilities (BPP)/Maximum Parsimony Bootstraps/Neighbor-Joining Bootstraps. Only nodes supported by more than two methods are indicated, the significant statistical supports being considered as higher than respectively 0.9/70/70. High support in individual gene trees was indicated: + for the γ-tub tree, × for the Ef1α tree, # for the β-tub tree. The tree is rooted based on previous studies (see text). Taxa labels correspond to the host plant on which fungal strains were collected. Brackets indicate clades and evolutionary units, *i.e. *cryptic fungal species, identified in the study using the three individual gene phylogenies (see text).

Our current findings lend additional support for the existence of a number of cryptic *Microbotryum *species, each specific to one or a few host species [29]. As before, the species status of the various clades rests on the congruence of the phylogenetic relationships of many strains inferred from the three fungal genes analyzed (Additional file 1, 2 and 3; see Methods for details on the inference of phylogenetic species). This congruence is only not met for the clade formed by the strains collected from *S. acaulis *as observed previously [29]. For these strains the γ-tub phylogeny differs from the two other single gene phylogenies (see AU tests in Table 2). The two placements of this lineage being basal, this incongruence is probably due to an ancient introgression or hybridization [29]. Without the *S. acaulis *strains there was no remaining incongruence: all supported nodes were identical in the three gene trees. We therefore concatenated the three genes in a dataset without the *S. acaulis *strains, yielding the same tree topology as in the individual phylogenies but with higher support for some nodes (Fig. 2).

**Table 2:** Results of the AU (Approximately Unbiased) tests for the *Microbotryum violaceum *dataset including strains from *Silene acaulis*.

		Topology enforced (MP tree with bootstraps > 70)		
		
		β-tub	γ-tub	Ef1α
Gene used	β-tub	0.995	0.002	0.346
	γ-tub	0.005	0.999	<0.001
	Ef1α	<0.001	0.002	0.681

*P*-values lower than 0.05 indicate that the likelihood of the topology obtained using one focal gene is significantly different from the likelihood of the enforced topologies (obtained using each other gene). When the *Microbotryum *strains parasitizing *S. acaulis *were removed in the fungal trees, all topologies were identical.

In addition to the cryptic species identified previously [29], three new phylogenetic species could be inferred from the strains analyzed in this study, respectively on *L. flos-jovis*, on *Saponaria officinalis*, and on *S. otites *(see Fig. 2 for species nomenclature extended from ref. [29]). We had access to only a single strain from *S. lemmonii *and *Stellaria *sp. plants, so we could not infer the existence of specific fungal phylogenetic species on these hosts. However, because these strains were both strongly separated from other identified phylogenetic species in their respective clades and because they were isolated from phylogenetically or geographically very distant hosts, we nevertheless considered them as separate lineages in the comparison of host and fungal phylogenies below. For *Stellaria*, this choice is in agreement with an ITS phylogeny supporting the monophyly of several strains collected from this genus with the same phylogenetic placement as ours [28].

On the 21 host species screened in the end we identified a total of 15 parasite species (Fig. 2). Most of them appeared specific to a single host species. For both *Atocion rupestris *(previously *Silene rupestris *[36]) and *Saponaria ocymoides *the two strains analyzed belonged to different fungal species. Because these hosts are seldom infected (pers. obs.) we suspect that we picked up transient opportunistic infections, so we did not consider these plant species as true hosts of the corresponding fungal species. The fungal species identified on the *Dianthus *species and on *Gypsophila repens *appeared generalist, *i.e. *able to infect several host species. Speciation on these hosts may alternatively be too recent to have allowed for sufficient molecular divergence for us to detect.

**Figure 3:**
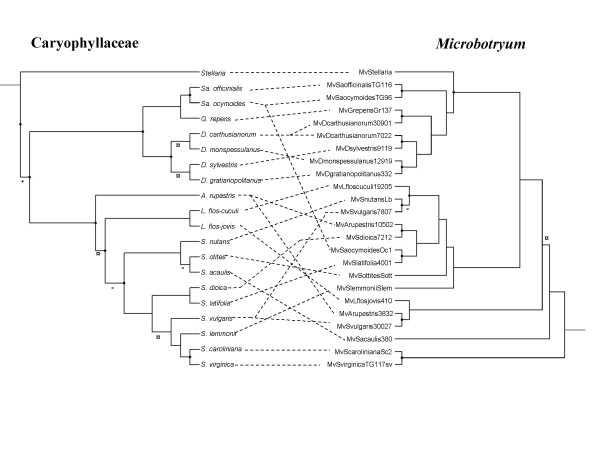
**Comparison of plant and fungal phylogenies using one strain per host species**. Representation of the associations between Caryophyllaceae (left) and *Microbotryum *(right) with the *a priori *least congruent combinations between all possible resolved topologies for host and parasite trees, using one strain per host species (with 'Min' topology for the plant tree, and 'A1' topology for fungal tree, see Table 3). The symbol * highlights resolution of a polytomy in the plant tree according to a previous study [33]; The symbol ¤ highlights resolutions in the plant tree and in the fungal tree that differed from the other topology tested (Max versus Min in plants, and 2 versus 1 for fungi); The symbol ^- ^highlights resolutions that had no impact on congruence between the two phylogenies. Dots on the nodes indicate where cospeciation events were inferred by TreeMap.

**Figure 4:**
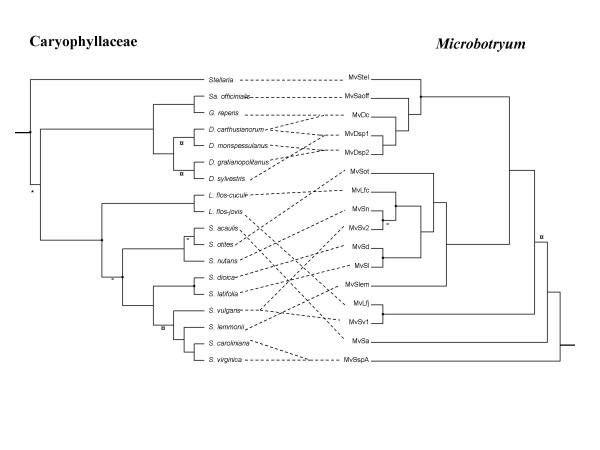
**Comparison of plant and fungal phylogenies using a single representative per fungal species**. Representation of the associations between Caryophyllaceae (left) and *Microbotryum *(right) with the *a priori *least congruent combinations between all possible resolved topologies for host and parasite trees, using a single representative per fungal species (with 'MinT' topology for the plant tree, and 'B1' topology for fungal tree, See Table 3). See Fig. 3 for symbol legend.

For some of the subsequent analyses (TreeMap, TreeFitter, Icong, see below and Methods), all nodes must be resolved. In that case, we used a previous study that obtained high supports to resolve one of our polytomies [33] (see symbol * on Figs. 3 and 4). For the remaining polytomies, we considered several alternative topologies (see legend of Tables 3, 4 and 5 for details and nomenclature) including the branch of the fungal species on *S. acaulis *for parasite phylogeny at the two alternative placements.

**Table 3:** Numbers of the different evolutionary events inferred in order to reconcile the plant and smut phylogenies by TreeMap (maximizing the number of cospeciation events).

Approach	Plant tree	Fungal tree	Cospeciation	Duplication	Host shift	Sorting events	Nb of events	P
Keeping one strain per host species	Max	A1	11	11	1	60	83	0.021*
		A2	12	11	0	58	81	0.041*
	Min	A1	11	12	0	68	91	0.023*
		A2	11	12	0	67	90	0.019*

Keeping a single representative per fungal species	MinT	B1	5	10	0	48	63	>0.5
		B2	5	10	0	46	61	>0.5
	MaxT	B1	6	9	0	43	58	0.287
		B2	6	9	0	41	56	0.268

Eight data sets were used. For the fungi, "A" trees contain one strain per smut species and per host (see Fig. 3) and "B" trees a single representative per smut species (see Fig. 4), and two possible topologies were used, "1" and "2", depending on the position of MvSa. For the plants, poorly supported nodes were resolved in order to maximize the congruence with the fungal trees in the "Max" trees and minimize it in the "Min" trees, and the "T" trees contain only host species on which fungal species are well established (*i.e. *ignoring the opportunistic infections). See also Figs. 3 and 4 for the correspondence between host and parasite phylogenies for the least congruent combinations.

**Table 4:** Mean numbers of the different evolutionary events inferred in order to reconcile the plant and smut phylogenies by TreeFitter.

Approach	Plant tree	Fungal tree	Costs	Cospeciation	Duplication	Host shift	Sorting events	Nb of events	Total cost	P
Keeping one strain per host species	Max	A1	C	6	0.5	16.5	2.5	25.5	32	0.0048**
			D	1	0	22	0	23	0	0.0371*
		A2	C	7	0.5	15.5	3.5	26.5	31	0.0042**
			D	1	0	22	0	23	0	0.0399*
	Min	A1	C	4	0	19	1	24	36	0.0430*
			D	1	0	22	0	23	0	0.0364*
		A2	C	5	0	18	2	25	35	0.0291*
			D	1	0	22	0	23	0	0.0434*

Keeping a single representative per fungal species	MinT	B1	C	3	0	12	2	17	30	0.0652
			D	0.5	0	14.5	0	15	0	0.3209
		B2	C	3	0	12	2	17	23	0.0903
			D	0.5	0	14.5	0	15	0	0.3355
	MaxT	B1	C	1	0	14	1.5	16.5	30	0.1937
			D	0	0	15	0	15	0	1
		B2	C	1	0	14	1.5	16.5	30	0.2331
			D	0	0	15	0	15	0	1

Eight data sets were used; see legend from Table 3 for their correspondence. Two sets of costs were used: C) maximizing the likelihood of cospeciation events: cospeciation = -1, duplication = 0, sorting events = 2, host shifts = 2; D) minimizing the number of events: cospeciation = 0, duplication = 0, sorting events = 2, host shifts = 0.

**Table 5:** Icong index and significance of the congruence level between Caryophyllaceae and *Microbotryum *trees.

Approach	Plant Tree	Fungal tree	Icong value	P
Keeping one strain per host species	MaxD	A1	1.400	0.007**
		A2	1.541	0.001****
	MinD	A1	1.260	0.057
		A2	1.260	0.057

Keeping a single representative per fungal species	MaxDS	B1	1.376	0.016*
		B2	1.549	0.001****
	MinDS	B1	1.204	0.175
		B2	1.376	0.016*

Eight data sets were used using fungal topologies described in Table 3. Plant trees had to be adjusted to be compatible with the fungal tree as the Icong program does not allow for multiple associations. In the name of the plant tree "D" indicates that hosts harbouring several fungal species were duplicated and "S" indicates that one host was chosen for generalist parasite species. These choices were made to maximize or minimize further the congruence with the fungal tree as compared to Max and Min trees (see legend Table 3). *P*-value < 0.05 indicated that the congruence between host and parasite trees was higher than that of two random trees.

### Comparison of plant and fungal phylogenies

We used two approaches for comparing host and parasite phylogenies: the more conventional one considered as a separate taxon the parasite strains from different host species such that generalist species were represented by as many terminal branches as host species on which they occurred (Fig. 3), while the second approach compared species phylogenies (retaining a single representative per fungal species) (Fig. 4). In both cases, some broad-scale congruence between the host and parasite phylogenies appears by visual inspection (the fungal phylogenetic species infecting the *Dianthus *and the *Saponaria *are monophyletic, as are the plants) while fine-scale congruence between the host and parasite phylogenies is weak, in particular among the parasites of *Silene*. However methodological analyses reconstructed different evolutionary histories for *Microbotryum-Caryophyllaceae *association depending on which approach was chosen.

When considering as a separate taxon the parasite strains from different host species (*i.e. *retaining one strain per host species and including opportunistic infections), all methods used for comparing these host and parasite phylogenies revealed a significant number of cospeciation events or of congruence level as compared to random associations. TreeMap [37], which seeks to minimize host shifts, inferred a significantly higher number of cospeciation events than expected from a random distribution but required many duplications and extinctions to achieve this (Table 3, upper half) and five distinct smut species were inferred on the ancestral host. The results were similar regardless of which topology was chosen for the unresolved nodes. Interestingly, TreeFitter [38] inferred a much higher number of host shifts than cospeciation, duplication and extinction events, and this was true regardless of the topology and the costs chosen (Table 4, upper half). The number of inferred cospeciation events was nonetheless higher than expected from a random distribution in all cases. ParaFit [39] detected a significant correlation between the plant and fungal trees (ParaFitGlobal = 0.0014, P < 0.0001 using the genetic distances and ParaFitGlobal = 0.7643, P < 0.0001 using the patristic distances). The Icong index [40] also indicated that the congruence between plant and fungal trees was significant or marginally significant (Table 5, upper half). Furthermore, we found a significant positive relationship between the genetic distances between pairs of host plants and the distance between the pairs of associated *Microbotryum *species using a Mantel test (a = 0.031, b = 0.125, P < 0.001).

The second approach, that we consider more correct, retains a single representative per fungal species, associated to each of the multiple hosts for generalist species (Fig. 4), which leads to ignore the opportunistic infections. This differs from the conventional approach in that it requires the proper delimitation of parasite species since species are not defined by the host, but rather as a lineage with an independent evolutionary history. In this case neither TreeMap (Table 3, bottom half) nor TreeFitter (Table 4, bottom half) inferred significantly more cospeciation events than expected from a random distribution, regardless of the chosen topologies for the unresolved nodes. For one of the two possible topologies of the plant phylogeny (MaxT, Table 4), TreeFitter even inferred only host shifts when costs where set to minimize the total number of events. Furthermore, the pairwise plant and *Microbotryum *genetic distances were not significantly correlated (Mantel test, a = 0.035, b = 0.064, P = 0.1173). Only the ParaFit and Icong analyses remained significant (ParaFitGlobal = 0.0014, P = 0.0002 using the genetic distances and ParaFitGlobal = 0. 7492, P = 0.00010 using the patristic distances; with Icong, P < 0.05 for three out of four of the combinations tested, Table 5, lower half), indicating that the parasite and host phylogenies were still more similar than expected by chance. The significant associations in the ParaFit results included both associations between *Dianthus *and *Saponaria *host species and their parasites, and some of the *Silene *species. The significance of these associations may be due to the symmetry of the two trees regarding the main clades: for instance, parasites found on *Dianthus *and *Saponaria *are monophyletic as are the plants.

We conclude that the failure to appropriately delimit parasite species and represent generalist parasite species as several separate taxa (one per host) had introduced a bias. Inspection of nodes for which reconciliation analyses supported cospeciation in TreeMap showed that the strains harboured by *Dianthus *spp. that belonged to the same generalist fungal species were considered as lineages arisen by cospeciation in the TreeMap reconciliation. In the ParaFit analyses, this same group of strains contributed almost half of the significant associations (data not shown). Thus, cospeciation events were inferred where there had been no speciation at all, but rather a failure to speciate or a host range expansion towards closely related hosts. Thus failure to delimit parasite species inflated the significance of congruence between the host and parasite trees.

### Geographic patterns

Most European host plant species used in this study have overlapping ranges. For instance, all the phylogenetic species, except those of the genus *Stellaria*, include at least one sample from the Western Alps and most of them at least one sample from the French Pyrenees (Additional file 4). The current geographic distribution of host species therefore appears to contribute little to the phylogenetic relationships among fungal species. In North America the hosts represent a clade whereas the smut sample collected from *S. lemmonii *was not related to smuts from the two other North American hosts (Fig. 2). Therefore we found no geographic pattern explaining the phylogenetic relationships of the fungal species.

### Relatedness among fungal species and host ecology

**Figure 5:**
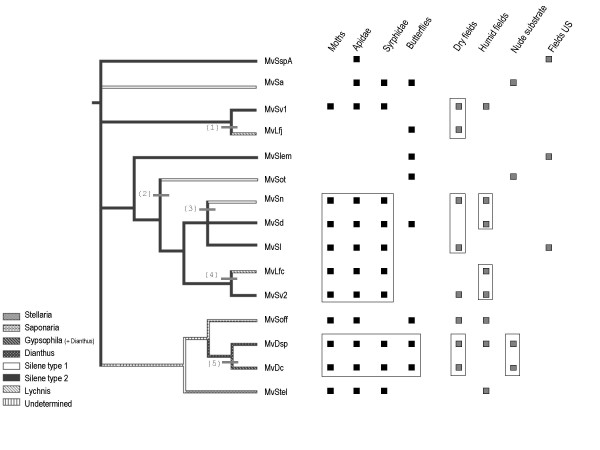
**Ecology of the host plants of the different *Microbotryum *species**. Phylogenetic tree of the *Microbotryum *species with indication, on the branches, of their plant host clade (see Fig. 1 for host clade delimitation). The numbers in brackets indicate the five terminal incongruences between the plant and fungal phylogenies. Here MvDsp1 and MvDsp2 were not distinguished (fused in MvDsp) because of poor resolution in this part of plant phylogeny and thus inability to identify any incongruence between the two phylogenies. Information about the ecology of the host plants is given: the most common clades of pollinators are indicated in black and type of habitat in grey (Fields from the U.S. were drawn apart to highlight the geographical barrier to host shift). Boxes indicate ecological similarities of host plants which parasite species are closely related.

In cases of host shifts, host ecology may play a role by facilitating contact between a parasite and a new host that has, for instance, similar pollinators or habitats as the original host. We therefore investigated whether recent host shifts, detected from non-congruence in the terminal branches of the host and parasite phylogenies (see Fig. 5), occurred between hosts with similar ecologies such as type of habitats and pollinator guilds. We detected several interesting ecological similarities among hosts with related smut lineages (see boxes on Fig. 5): the pair *S. vulgaris*/*L. flos-jovis *grows on well drained soils, such as calcareous meadows, at least in the Western Alps, which could have facilitated potential host shift 1. Sphingidae, Noctuidae (in particular *Hadena bicruris*), Apidae and Syrphidae all visit *Silene nutans, L. flos-cuculi*, *S. vulgaris, S. latifolia *and *S. dioica *and may thus have been the agents of host shifts 3 and 4. Regarding potential host shift 4, *Lychnis flos-cuculi*, *S. vulgaris*, as well as *S. nutans *are often found together on shady borders between fields and woods. The *Dianthus *spp. and *G. repens *both grow on exposed rocky areas and are pollinated by Syrphidae, potentially favouring host shift 5. Intriguingly, *S. otites *shares little similarities with other *Silene *from which it could have inherited its parasites. It is mainly wind-pollinated, only shares butterflies as insect pollinators with *S. lemmonii *which could have facilitated host shift 2, but these two plant species have no current geographical overlap to our knowledge. This suggests that the incongruences 1, 3, 4, and 5 (Fig. 5) between host and parasite phylogenies, if indeed the results of host shifts, may have been facilitated by ecological similarities of the plants. However, the lack of specificity of insect pollinators, the large overlap of the ecotypes of the different hosts and the probable rapid evolution of ecological traits at the examined scale render any such conclusions speculative.

## Discussion

### Caryophylloideae phylogeny

The host phylogeny, here including 20 species within the sub-family Caryophylloideae provides interesting insights in itself, because the relationships among Caryophyllaceae are still poorly known, in particular at the sub-genus level [32-34]. It is striking that the three North American hosts appear monophyletic, branching within the European species. This would be consistent with their evolution by allopatric speciation following a single colonization event from Europe, after the separation of these two continents. Fossil records suggest an origin of the Caryophyllaceae 70 Mya in Australia, the oldest fossils in Europe dating back only 30–50 Mya [35], by which time North America was separated from Europe by the Atlantic Ocean [41].

Our dataset, with additional species compared to previous studies [33,35], further supports the monophyly of the genera *Lychnis*, *Saponaria *and *Dianthus *but unfortunately fails to resolve the monophyly of the genus *Silene*. The relative position of species within the *Dianthus *clade was the least resolved, suggesting recent radiation of *Dianthus *species. This may be particularly relevant for the study of *Microbotryum*-Caryophyllaceae association because the smut lineages on *Dianthus *species also appear to be less host-specific than those on *Silene *species.

### Importance of delimiting parasite species in cophylogeny studies

We detected a number of cryptic generalist species with multiple closely related hosts. When these were duplicated in the phylogeny to have one representative per host species, the congruence between the host and parasite phylogenies was artificially inflated. This shows that carefully delimiting parasite species boundaries in cophylogeny studies is important for assessing the degree of cophylogenetic history, although it is rarely done for micro-organisms. Most often parasites are simply sampled from different host species and the resulting tree is compared to the tree for host species [9,14]. Such an approach implicitly assumes that each host species harbours one distinct parasite species, an assumption that we here show to be both unfounded and of potentially great importance in this kind of study. The *Microbotryum*-Caryophyllaceae system shows further that generalist parasite species are likely to infect a range of closely related hosts. Therefore parasite species boundaries must be carefully delimited to avoid erroneous interpretations of the degree of congruence between host and parasite phylogenies.

### Inference on *Microbotryum *evolutionary history: host shifts versus cospeciation

After having carefully delimited fungal species, the number of cospeciation events inferred by TreeMap and Tree Fitter when reconciling the *Microbotryum *phylogeny to that of their Caryophyllaceaous hosts was not higher than expected from a random distribution, as suggested by a previous study on a smaller sample [14] nor was there a significant correlation (Mantel test) between genetic distances of host plant and their respective anther smut species.

Icong and ParaFit still detected significant congruence between the host and fungal phylogenies, the significant associations in ParaFit being mostly due to the parasite species found on *Dianthus *and *Saponaria*. The significance of the global ParaFit and Icong tests therefore most probably stems, not from pervasive cospeciation events, but from the fact that the anther smut lineages infecting the *Dianthus *and *Saponaria *plants were monophyletic, as were the plants. This pattern is consistent with cospeciation or host shifts between closely related members of these clades, together with an absence of host shifts between distantly related hosts like *Silene *and *Dianthus*. Host shifts appear to have occurred between hosts with limited phylogenetic distances in the *Microbotryum*-Caryophyllaceae disease system. Incipient host shifts by *Microbotryum *have been reported onto *S. vulgaris *from *S. latifolia *[18,42] and onto *Gypsophila repens *from the closely related *Petrorhagia saxifraga *[43]. The large-scale congruence detected by ParaFit and Icong is thus likely to be due to constraints impeding shifts to hosts that are genetically too distant. Investigating the branch lengths and divergence times of the two phylogenies would be necessary to determine whether cospeciation occurred at all [44]. Unfortunately, available fossil records do not allow calibration of the sequence divergence between the *Microbotryum *and its host plant species [35,45].

Overall, our results suggest that cospeciation is not the rule in the *Microbotryum*-Caryophyllaceae system, that host shifts were pervasive, but that fungal species could not shift to too distant host species (shift either associated with speciation or not). Comparing the results of reconciliation analyses to that of methods investigating simple congruence was important for identifying these constraints on host shifts imposed by host phylogeny.

Several factors may have limited the power of our analyses on cophylogeny. First, our collection was restricted to the most common Caryophyllaceae: we have sampled 21 host species of the more than 100 known to be infected with anther smut [17]. Several additional independent fungal lineages that may show more evidence of cospeciation than the ones we have sampled are therefore likely to exist. However, larger sampling is more likely to decrease and not increase the global congruence between hosts and parasites if host shifts occur between moderately distant hosts as shown for *Microbotryum *species on *Silene*. And indeed larger sampling gave evidence for host shifts in the highly specific ant-fungus growing parasites (compare conclusions of references [46] and [47]). Our conclusion that *Microbotryum *has mainly evolved by host shifts is therefore highly likely to hold with a larger sampling. Second, the lineages that appear to infect more than one host species could actually be host-specific but too recently divergent for our markers to detect differentiation. This would increase the congruence of the *Microbotryum *and Caryophyllaceae phylogenies, but would not decrease the high number of host shifts required to reconcile the two phylogenies. It would thus not affect our conclusions regarding *M. violaceum *evolutionary history: the *Microbotryum*-Caryophyllaceae system is another example of a host-parasite association where cocladogenesis is not the rule and highlights the importance of cross species disease transmission in the emergence of new parasites lineages [9,11-14].

## Conclusion

Adaptive specialization that follows rare host shift events appears to be the major mechanism of speciation in *Microbotryum*, as in many pathogenic fungi [4,48,49]. The frequency of host shifts in the *Microbotryum*-Caryophyllaceae system, and in several other highly specific plant-parasite associations [9,13,14], illustrates that specialization is far from an evolutionary dead end and that the diversification of specialist species can occur by other phenomena than cospeciation. In agreement with this idea, most previous cophylogeny studies on parasites able to disperse to novel hosts have reported evidence for host shifts, even when the number of cospeciation events was found significant [47,50]. The possibility of host shift and the degree of relatedness between ancestral and new hosts will *a priori *depend on several factors, such as geography, ecology, and the genetics of specialization. Current geographic distribution of host species seems to be of little relevance for understanding the association between *Microbotryum *and its hosts at a local or regional scale. We detected some interesting examples of shared ecological traits that may have facilitated host shifts.

## Methods

### Taxon Sampling

To test whether there is a phylogenetic association between anther smut fungi and their Caryophyllaceae hosts, we used data from 21 host species from Europe and North America for which we have observed *Microbotryum *infections in natural populations. All host species analyzed in this study belong to subfamily Caryophylloideae, except one *Stellaria *species, belonging to the subfamily Alsinoideae [33]. To root host phylogenetic trees, the *Stellaria *sp. was used as an outgroup based on a previous molecular phylogenetic study [33]. To root anther smut phylogenetic trees, the strains from the North American hosts *S. caroliniana *and *S. virginica *were used because they were previously shown to branch at the base of all the species analyzed in the present study [28]. Because the plant species are reasonably well established [31], a single plant sample was collected per host species. Some North American *Silene *species are not monophyletic [51] but they were not those included in our dataset. Several fungal samples per host species were collected whenever possible because the parasite taxonomy is currently being resolved (see above). The origins of anther smut and host samples are given in the Additional files 4 and 5, respectively.

Infected plants were detected by the violet, sporulating anthers of open flowers and flower buds from these same plants were collected and stored in individual paper or glacine envelopes on silica gel.

### Molecular Methods

Host plant DNA extraction and PCR amplification of plant nuclear ITS and cpDNA (the intron within the *trn*L gene, hereafter *trn*L, and the intergenic region between the genes *trn*L and *trn*F, hereafter *trn*LF) were performed as described previously [52]. DNA from the fungal strains was extracted from the cultures using a Chelex protocol [53]. Fungal DNA extraction and PCR amplification of the β-tubulin (β-tub), γ-tubulin (γ-tub) and Elongation factor 1 α (Ef1α) were amplified according to [29]. PCR fragments were purified and sequenced as described previously [29,52].

The programs Navigator PPC (Applied Biosystems) and Bioedit 6.0.7 [54] were used to check sequence electropherograms. Multiple alignments based on consensus sequences were carried out using BioEdit. Alignments were then checked and apparent alignment errors were corrected by hand. Regions of ambiguous alignment and gaps were excluded from all analyses.

### Sequence data

The sequences generated are available in GenBank (Accession numbers plant ITS are EF407925–EF407945, for plant *trn*L EF407883–EF407903, for plant *trn*LF EF407904–EF407924, for fungal β-tub, DQ992076–DQ992113 and EF419304, γ-tub, DQ992114–DQ992147, and Ef1α, DQ992148–DQ992177 and EF419301–EF419303). The smut sequences generated previously [29] were also used, and are indicated in the Additional file 4.

### Phylogenetic Analysis

Phylogenetic trees were reconstructed by Bayesian inference, maximum parsimony (MP) and Neighbor-Joining (NJ). MP and NJ analyses were performed using PAUP version 4.0b10 [55]. The following options were employed for MP analyses in PAUP: heuristic search, characters unordered with equal weight, starting tree obtained via stepwise addition option and constructed with random sequence addition (10 replicates), branch swapping by TBR (tree bisection reconnection). A single MP tree was recovered for all datasets. NJ analyses were performed using the molecular evolution models selected by AIC in ModelTest 3.7 [56]. The models retained were TIM+G, gamma shape = 0.41 for the concatenated plant data set, HKY+G, Ti/Tv = 2.2, gamma shape = 1.12 for the fungal β-tub gene, HKY+I, invariable sites = 0.52 for the fungal γ-tub gene, GTR+G, gamma shape = 0.24 for the fungal Ef1α gene and GTR+G, gamma shape = 0.26 for the concatenated fungal dataset. Bootstrap confidence values were calculated for 1,000 pseudoreplicates. Bayesian analyses were run using MrBayes version 3.0b5 [57]. Each run consisted of 4 incrementally heated Markov chains run simultaneously, with heating value set to default (0.2). Priors were constrained according to the results obtained by running MrModeltest 2.2 [58]. Markov chains were initiated from a random tree and run for increasing numbers of generations, until the average standard deviation remained below 0.01, *i.e. *1,000,000 generations for Ef1α, 1,250,000 generations for γ-tub, 1,000,000 generations for β-tub, 500,000 generations for the fungal and plant concatenated datasets. Trees were sampled every 50 generations and the first 25% of trees were not taken into account. We used a 50% majority rule consensus tree to obtain the Bayesian posterior probabilities (Bpp). Details on the phylogenetic parameters used and output statistics are available upon request. Data matrices and resulting trees are available in TreeBase (submission ID number SN3239; Journal Peer Reviewer's PIN number: 30601). We considered nodes as strongly supported by a given method when they had values of Bayesian Posterior Probabilities/Maximum Parsimony Bootstraps/Neighbor-Joining Bootstraps at least equal to 0.9/70/70, respectively. Monophyly supported by at least two methods was considered as significant.

### Congruence between individual phylogenies within fungal or plant systems

Congruence between individual phylogenies was estimated by Approximately Unbiased tests (AU) as implemented in CONSEL [59], by comparing for each gene the likelihood of the MP topology obtained for this gene to the likelihood of the enforced topologies (obtained with each other gene) [60]. Likelihoods were obtained in PAUP using the sequence evolution model selected, using AIC, according to the results of ModelTest v. 3.7 [56]. The incongruence length difference test (ILD, [61]) was not used because several works have underlined that the ILD test is a poor indicator of data set combinability [62].

In absence of significant difference, we further checked the congruence of each node by visual inspection. Nodes were considered as congruent in two gene phylogenies when supported by significant statistical values of at least two of the three phylogenetic reconstruction methods in each of the two phylogenies. Nodes were considered as incongruent between two gene phylogenies when significant statistical values of at least two of the three phylogenetic reconstruction methods supported conflicting nodes between the two gene phylogenies.

When we found no evidence for incongruence, the genes were concatenated to perform combined analyses, similarly as described above. Consistency between the resulting tree and individual gene trees was again checked by visual inspection.

### Identification of fungal phylogenetic species

To detect phylogenetic species within *M. violaceum*, we used the criterion of phylogenetic congruence between different gene phylogenies [29]. We thus considered a group of strains as an independent evolutionary lineage when 1) it was strongly supported as monophyletic by two of the three reconstruction methods in at least one gene phylogeny or in the concatenated phylogeny, and 2) this was not contradicted by the other gene phylogenies. Using three different methods of reconstruction allows us to be conservative in our species delimitation rule, and to avoid splitting the fungus into too many species based on some artefact of one particular method. We considered here again nodes as strongly supported by a given method when they had values of Bayesian Posterior Probabilities/Maximum Parsimony Bootstraps/Neighbor-Joining Bootstraps at least equal to 0.9/70/70, respectively.

### Comparison of host and fungal trees

To compare the plant and fungal phylogenies, we used the data derived from the concatenated sequences. As we wanted to assess the impact of species delimitation, we retained successively: 1) one fungal strain per host species, and 2) one fungal strain per fungal species but linking it to all the hosts that this parasite species was found to infect.

For reconciliation analyses (TreeMap [37] and TreeFitter [38]) and Maximum Agreement Subtrees (Icong index [40]), which do not accept polytomies, phylogenetic relationships in our plant tree that were poorly supported were resolved when possible according to previous studies (see symbol * on Figs. 3 and 4). For the remaining unresolved nodes, the alternative placements were considered as equally possible. In order to reduce the combinations of plant and fungal tree comparisons, we used only two topologies among all possible ones, for each of the plants and fungi. One topology was chosen as *a priori *maximizing the congruence with the other partner and the other one minimizing it (see symbol ¤ on Fig. 3 and 4). For the methods using genetic distances (Mantel tests) or patristic distances (ParaFit) original datasets were conserved but we excluded *S. acaulis*, because of the incongruence between individual gene phylogenies, which may have resulted from hybridization between two distant lineages.

The history of association between hosts and fungi was first investigated using reconciliation analysis as implemented in the programs TreeMap [37] and TreeFitter [38]. TreeMap 1 uses a model to find optimal reconstructions of the history of the association by maximizing cospeciation events [37]. In situations when host shifts are likely to be common, as in our case [14], this methodology is less likely to find optimal solutions than when host shifts are rare [63]. A later release of this program, TreeMap 2.0b [64], considers all potentially optimal solutions and offers a more appropriate means of dealing with host shifts. However, this program is currently limited in size and complexity of datasets that can be computed, and we could not run the complete analysis with our dataset. Thus, in this study we used the program TreeMap 1 with the heuristic search option to reconcile plant and fungal trees. The program TreeFitter 1.0 uses a different algorithm and optimality criterion for reconciling the host and fungal phylogenies, allowing reconstructions involving many host shifts to be recovered. In both TreeMap and TreeFitter, one can test the null hypothesis that the two phylogenies are randomly related by comparing the scores of optimal reconstructions (*i.e. *the number of cospeciation events for TreeMap and global cost for TreeFitter) with those of randomly obtained phylogenies through permutational procedures. We chose to randomize the parasite trees because in cophylogeny analyses, the host tree is considered as given, and one wants to test whether the observed parasite tree is more congruent with the host tree than are random parasite trees. Tests were performed based on 3000 permutations for TreeMap and 10,000 permutations for TreeFitter. TreeFitter allows assignment of different costs to four types of events (*i.e. *cospeciation, duplication, extinction, and host shift). When using the option seeking for costs with the highest likelihood, we found a very large range of possible costs. We varied these costs to assess effects on the test results, and retained one set of costs maximizing the likelihood of cospeciation events inferred, and the other one minimizing the total number of events.

As a third approach we used the method ParaFit [39] that uses matrices of principal coordinates, derived either from patristic distances (summed branch lengths along a phylogenetic tree; in that case, *S. acaulis *was excluded) or genetic distances, and the matrix of presence/absence of host parasite associations. Using patristic distances allows taking into account the most likely phylogenetic relationships in addition to genetic distances. We performed both analyses. The trace statistic is calculated by taking plant-fungus associations into account. The null hypothesis that the plant and fungal samples are randomly associated is tested by a permutational procedure. Patristic distances were calculated from the unresolved trees derived from the concatenated datasets using PATRISTIC [65,66]. Phylogenetic distances were calculated using the software MEGA [67]. Principal coordinates were then computed using the software DistPCoA [68].

The fourth method assessed whether the plant and fungal topologies were more similar than expected by chance using the Icong index [40]. With this method, the topological congruence of two trees is assessed through their maximum agreement subtree (MAST). A MAST is the largest possible tree compatible with two given trees [69] and is obtained by removing the minimum number of leaves (*i.e. *terminal branches) in both trees in order to obtain perfect congruence. Significant congruence is inferred when congruence between the two trees is higher than that of random trees with the same leaf number. As for the previous methods, we reduced the number of topologies tested by choosing two topologies for the plant tree and two for the fungal tree. As this method requires that the trees had the same number of leaves, hosts harbouring several fungal species were duplicated. When using a single strain per fungal species, we also had to select a single one of the multiple hosts. The choice was made so that the congruence with the fungal tree was maximized or minimized. We assessed the level of congruence for the four resulting combinations. Finally, we correlated genetic distances of host plants with the distances between associated *Microbotryum *species. Genetic distances were computed as for ParaFit. As the data were not independent, we tested the matrix correlation using permutations (Mantel test in the software Genepop [70,71]).

### Pollinator and ecological data

Data on the common pollinators of the Caryophyllaceae species included in this study were collected from the literature [27,72-77] and from personal observations and communications (J.A. Shykoff, A. Erhardt). Some individual species, e.g. *Hadena bicruris*, were reported as pollinators, but most descriptions were made at higher taxonomic levels (e.g. genus or family) or common name (e.g. moths or butterflies). This raises the concern that plants pollinated by the same genus or family may not be visited by exactly the same species or the same individuals. For well studied plant species, however, only a few different pollinators are reported and these pollinator species are rather unspecialized, being found pollinating several plant genera or families [27,72,74]. For instance, *Macroglossum stellatarum *(Sphingidae), *Hadena bicruris*, and *Autographa gamma *(Noctuidae) were all found on at least three different host species [72]. Moreover, other observations showed that individual pollinators land successively on three sympatric species: *Dianthus carthusianorum*, *Silene vulgaris *and *Silene nutans *[27]. We therefore considered that sharing a clade of pollinators implied sharing at least one pollinator species, thus possibly allowing *M. violaceum *spore transmission.

To partition the plant species into ecological groups based on their most common habitats, the French botanical web site SOPHY [78], literature data [79], and personal observations and communications (J.A. Shykoff, A. Erhardt, C. Bock) were used. Ecological similarity may lead to cross-species disease transmission even in the absence of shared insect pollinators because spores can also be disseminated by wind, rain and phytophageous insects [27].

## Authors' contributions

TG, JAS and AW contributed to the conception and design of the study, to the acquisition and analysis of data, to coordination of the study, and were involved in drafting the manuscript. GR, FJ, MLG, MEH and RY participated in the acquisition and analysis of data, and in drafting of the manuscript. All authors read and approved the final manuscript.

## Supplementary Material

Additional file 1 **Bayesian 50% majority-rule consensus tree of the *Microbotryum *strains analyzed in this study based on the γ-tub gene**. Statistical supports indicate Bayesian Posterior Probabilities (BPP)/Maximum Parsimony Bootstraps/Neighbor-Joining Bootstraps. Only nodes supported by more than two methods are indicated, the significant statistical supports being considered as higher than respectively 0.9/70/70. The tree is rooted based on previous studies (see text). Taxa labels correspond to the host plant on which fungal strains were collected. Clades not supported in the individual tree are indicated in grey. Click here for file

Additional file 2 **Bayesian 50% majority-rule consensus tree of the *Microbotryum *strains analyzed in this study based on the Ef1α gene**. Statistical supports indicate Bayesian Posterior Probabilities (BPP)/Maximum Parsimony Bootstraps/Neighbor-Joining Bootstraps. Only nodes supported by more than two methods are indicated, the significant statistical supports being considered as higher than respectively 0.9/70/70. The tree is rooted based on previous studies (see text). Taxa labels correspond to the host plant on which fungal strains were collected. Clades not supported in the individual tree are indicated in grey. Click here for file

Additional file 3 **Bayesian 50% majority-rule consensus tree of the *Microbotryum *strains analyzed in this study based on the β-tub gene**. Statistical supports indicate Bayesian Posterior Probabilities (BPP)/Maximum Parsimony Bootstraps/Neighbor-Joining Bootstraps. Only nodes supported by more than two methods are indicated, the significant statistical supports being considered as higher than respectively 0.9/70/70. The tree is rooted based on previous studies (see text). Taxa labels correspond to the host plant on which fungal strains were collected. Clades not supported in the individual tree are indicated in grey. Click here for file

Additional file 4 Host species, name and sampling localities of the *Microbotryum *smut fungi analysed in this study. Click here for file

Additional file 5 Sampling localities of plant samples analysed in this study. Click here for file
