# Supplementary material for: Cophylogeny of the anther smut fungi and their caryophyllaceous hosts: Prevalence of host shifts and importance of delimiting parasite species for inferring cospeciation
Source: BMC Evol Biol. 2008 Mar 27;8:100. doi: 10.1186/1471-2148-8-100 (PMC2324105; doi:10.1186/1471-2148-8-100)
Supplement: Additional file 1 — Bayesian 50% majority-rule consensus tree of the Microbotryum strains analyzed in this study based on the γ-tub gene. Statistical supports indicate Bayesian Posterior Probabilities (BPP)/Maximum Parsimony Bootstraps/Neighbor-Joining Bootstraps. Only nodes supported by more than two methods are indicated, the significant statistical supports being considered as higher than respectively 0.9/70/70. The tree is rooted based on previous studies (see text). Taxa labels correspond to the host plant on which fungal strains were collected. Clades not supported in the individual tree are indicated in grey. [file 1471-2148-8-100-S1.ppt]

## Slide 1
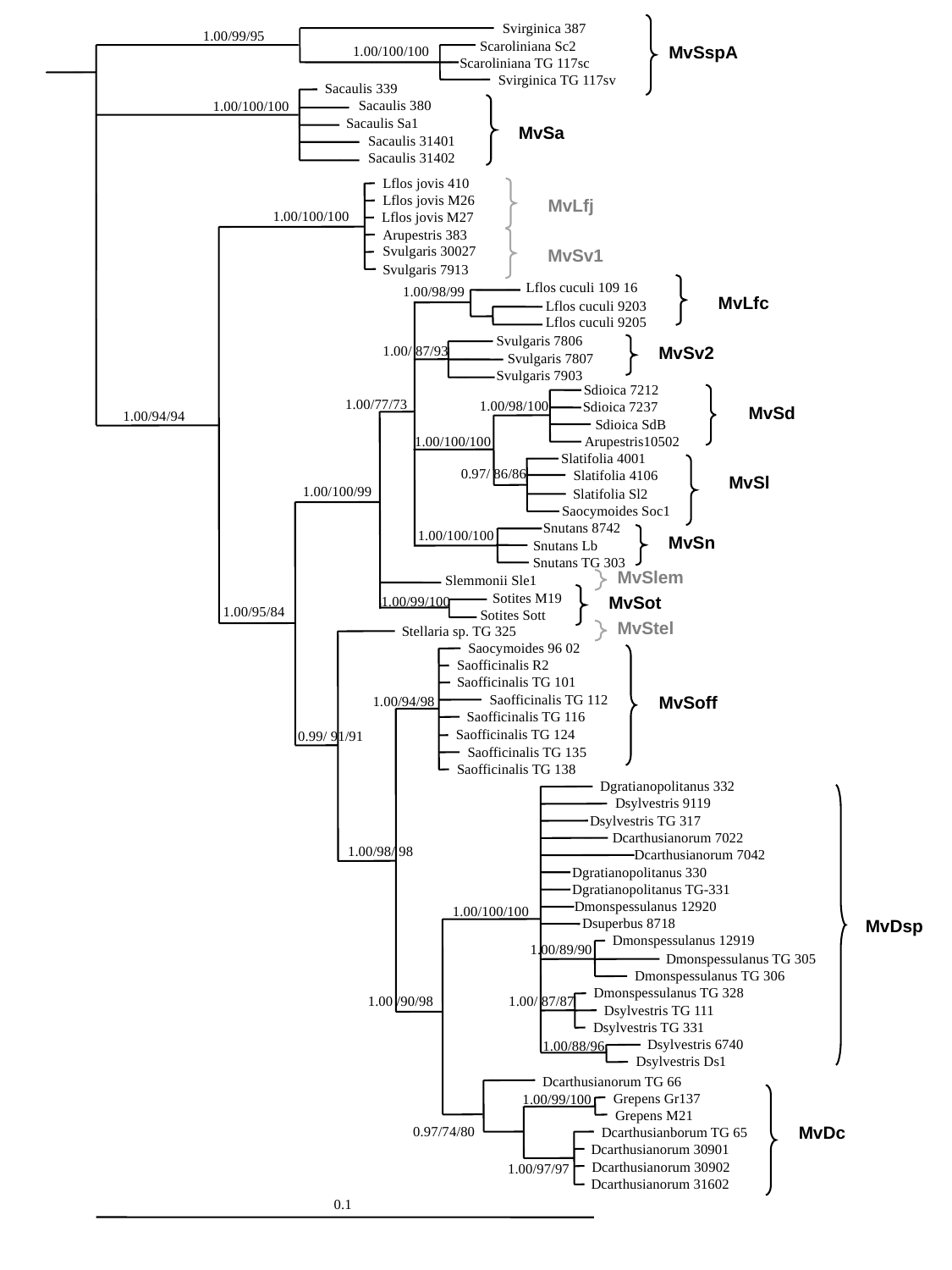

Svirginica 387
1.00/99/95
MvSspA
Scaroliniana Sc2
1.00/100/100
Scaroliniana TG 117sc
Svirginica TG 117sv
Sacaulis 339
Sacaulis 380
1.00/100/100
Sacaulis Sa1
MvSa
Sacaulis 31401
Sacaulis 31402
Lflos jovis 410
MvLfj
Lflos jovis M26
1.00/100/100
Lflos jovis M27
Arupestris 383
MvSv1
Svulgaris 30027
Svulgaris 7913
Lflos cuculi 109 16
1.00/98/99
MvLfc
Lflos cuculi 9203
Lflos cuculi 9205
Svulgaris 7806
MvSv2
1.00/ 87/93
Svulgaris 7807
Svulgaris 7903
Sdioica 7212
1.00/77/73
MvSd
1.00/98/100
Sdioica 7237
1.00/94/94
Sdioica SdB
1.00/100/100
Arupestris10502
Slatifolia 4001
0.97/ 86/86
MvSl
Slatifolia 4106
1.00/100/99
Slatifolia Sl2
Saocymoides Soc1
Snutans 8742
MvSn
1.00/100/100
Snutans Lb
Snutans TG 303
MvSlem
Slemmonii Sle1
MvSot
Sotites M19
1.00/99/100
1.00/95/84
Sotites Sott
MvStel
Stellaria sp. TG 325
Saocymoides 96 02
Saofficinalis R2
Saofficinalis TG 101
MvSoff
Saofficinalis TG 112
1.00/94/98
Saofficinalis TG 116
Saofficinalis TG 124
0.99/ 91/91
Saofficinalis TG 135
Saofficinalis TG 138
Dgratianopolitanus 332
Dsylvestris 9119
Dsylvestris TG 317
Dcarthusianorum 7022
1.00/98/ 98
Dcarthusianorum 7042
Dgratianopolitanus 330
Dgratianopolitanus TG-331
Dmonspessulanus 12920
1.00/100/100
MvDsp
Dsuperbus 8718
Dmonspessulanus 12919
1.00/89/90
Dmonspessulanus TG 305
Dmonspessulanus TG 306
Dmonspessulanus TG 328
1.00 /90/98
1.00/ 87/87
Dsylvestris TG 111
Dsylvestris TG 331
Dsylvestris 6740
1.00/88/96
Dsylvestris Ds1
Dcarthusianorum TG 66
Grepens Gr137
1.00/99/100
Grepens M21
MvDc
0.97/74/80
Dcarthusianborum TG 65
Dcarthusianorum 30901
Dcarthusianorum 30902
1.00/97/97
Dcarthusianorum 31602
0.1
