# Supplementary material for: Cophylogeny of the anther smut fungi and their caryophyllaceous hosts: Prevalence of host shifts and importance of delimiting parasite species for inferring cospeciation
Source: BMC Evol Biol. 2008 Mar 27;8:100. doi: 10.1186/1471-2148-8-100 (PMC2324105; doi:10.1186/1471-2148-8-100)
Supplement: Additional file 2 — Bayesian 50% majority-rule consensus tree of the Microbotryum strains analyzed in this study based on the Ef1α gene. Statistical supports indicate Bayesian Posterior Probabilities (BPP)/Maximum Parsimony Bootstraps/Neighbor-Joining Bootstraps. Only nodes supported by more than two methods are indicated, the significant statistical supports being considered as higher than respectively 0.9/70/70. The tree is rooted based on previous studies (see text). Taxa labels correspond to the host plant on which fungal strains were collected. Clades not supported in the individual tree are indicated in grey. [file 1471-2148-8-100-S2.ppt]

## Slide 1
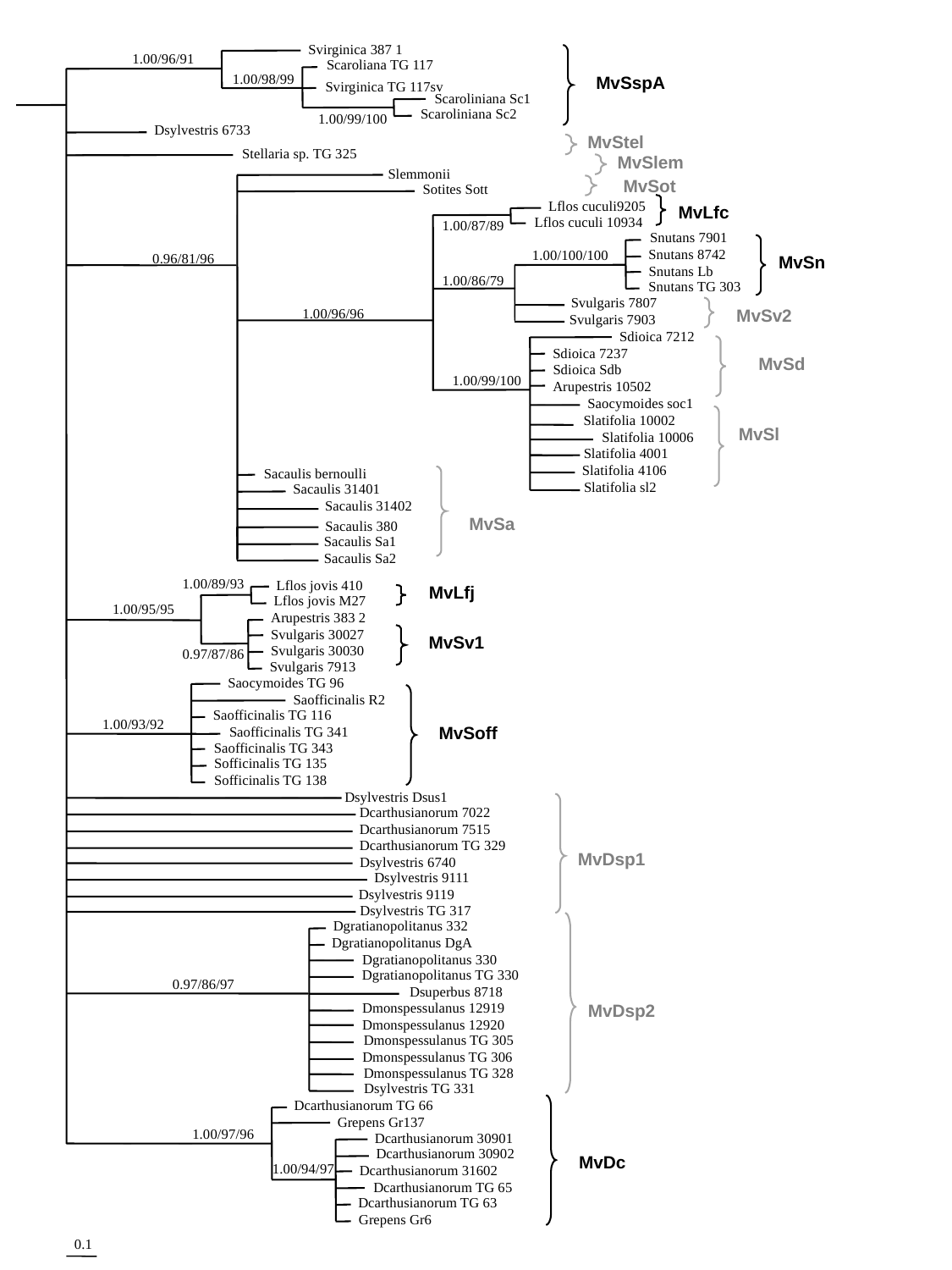

Svirginica 387 1
1.00/96/91
Scaroliana TG 117
MvSspA
1.00/98/99
Svirginica TG 117sv
Scaroliniana Sc1
Scaroliniana Sc2
1.00/99/100
Dsylvestris 6733
MvStel
MvSlem
Stellaria sp. TG 325
Slemmonii
MvSot
Sotites Sott
MvLfc
Lflos cuculi9205
Lflos cuculi 10934
1.00/87/89
Snutans 7901
MvSn
Snutans 8742
1.00/100/100
0.96/81/96
Snutans Lb
1.00/86/79
Snutans TG 303
Svulgaris 7807
MvSv2
1.00/96/96
Svulgaris 7903
Sdioica 7212
Sdioica 7237
MvSd
Sdioica Sdb
1.00/99/100
Arupestris 10502
Saocymoides soc1
Slatifolia 10002
MvSl
Slatifolia 10006
Slatifolia 4001
Slatifolia 4106
Sacaulis bernoulli
Slatifolia sl2
Sacaulis 31401
Sacaulis 31402
MvSa
Sacaulis 380
Sacaulis Sa1
Sacaulis Sa2
1.00/89/93
MvLfj
Lflos jovis 410
Lflos jovis M27
1.00/95/95
Arupestris 383 2
MvSv1
Svulgaris 30027
Svulgaris 30030
0.97/87/86
Svulgaris 7913
Saocymoides TG 96
Saofficinalis R2
Saofficinalis TG 116
1.00/93/92
MvSoff
Saofficinalis TG 341
Saofficinalis TG 343
Sofficinalis TG 135
Sofficinalis TG 138
Dsylvestris Dsus1
Dcarthusianorum 7022
Dcarthusianorum 7515
Dcarthusianorum TG 329
MvDsp1
Dsylvestris 6740
Dsylvestris 9111
Dsylvestris 9119
Dsylvestris TG 317
Dgratianopolitanus 332
Dgratianopolitanus DgA
Dgratianopolitanus 330
Dgratianopolitanus TG 330
0.97/86/97
Dsuperbus 8718
MvDsp2
Dmonspessulanus 12919
Dmonspessulanus 12920
Dmonspessulanus TG 305
Dmonspessulanus TG 306
Dmonspessulanus TG 328
Dsylvestris TG 331
Dcarthusianorum TG 66
Grepens Gr137
1.00/97/96
Dcarthusianorum 30901
Dcarthusianorum 30902
MvDc
1.00/94/97
Dcarthusianorum 31602
Dcarthusianorum TG 65
Dcarthusianorum TG 63
Grepens Gr6
0.1
