# Supplementary material for: Cophylogeny of the anther smut fungi and their caryophyllaceous hosts: Prevalence of host shifts and importance of delimiting parasite species for inferring cospeciation
Source: BMC Evol Biol. 2008 Mar 27;8:100. doi: 10.1186/1471-2148-8-100 (PMC2324105; doi:10.1186/1471-2148-8-100)
Supplement: Additional file 3 — Bayesian 50% majority-rule consensus tree of the Microbotryum strains analyzed in this study based on the β-tub gene. Statistical supports indicate Bayesian Posterior Probabilities (BPP)/Maximum Parsimony Bootstraps/Neighbor-Joining Bootstraps. Only nodes supported by more than two methods are indicated, the significant statistical supports being considered as higher than respectively 0.9/70/70. The tree is rooted based on previous studies (see text). Taxa labels correspond to the host plant on which fungal strains were collected. Clades not supported in the individual tree are indicated in grey. [file 1471-2148-8-100-S3.ppt]

## Slide 1
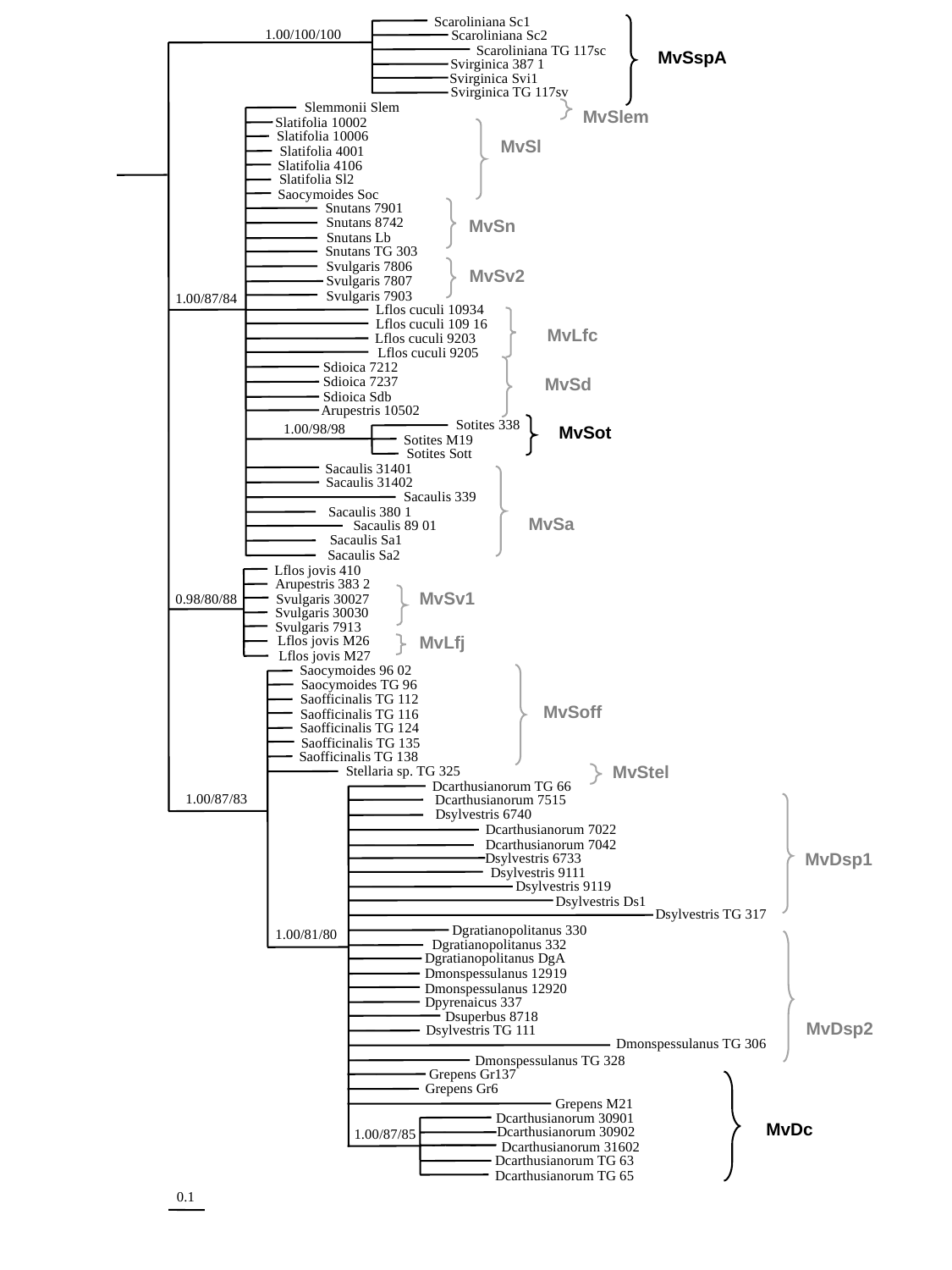

Scaroliniana Sc1
1.00/100/100
Scaroliniana Sc2
MvSspA
Scaroliniana TG 117sc
Svirginica 387 1
Svirginica Svi1
Svirginica TG 117sv
Slemmonii Slem
MvSlem
Slatifolia 10002
Slatifolia 10006
MvSl
Slatifolia 4001
Slatifolia 4106
Slatifolia Sl2
Saocymoides Soc
Snutans 7901
MvSn
Snutans 8742
Snutans Lb
Snutans TG 303
Svulgaris 7806
MvSv2
Svulgaris 7807
Svulgaris 7903
1.00/87/84
Lflos cuculi 10934
Lflos cuculi 109 16
MvLfc
Lflos cuculi 9203
Lflos cuculi 9205
Sdioica 7212
MvSd
Sdioica 7237
Sdioica Sdb
Arupestris 10502
MvSot
Sotites 338
1.00/98/98
Sotites M19
Sotites Sott
Sacaulis 31401
Sacaulis 31402
Sacaulis 339
Sacaulis 380 1
MvSa
Sacaulis 89 01
Sacaulis Sa1
Sacaulis Sa2
Lflos jovis 410
Arupestris 383 2
MvSv1
Svulgaris 30027
0.98/80/88
Svulgaris 30030
Svulgaris 7913
MvLfj
Lflos jovis M26
Lflos jovis M27
Saocymoides 96 02
Saocymoides TG 96
Saofficinalis TG 112
MvSoff
Saofficinalis TG 116
Saofficinalis TG 124
Saofficinalis TG 135
Saofficinalis TG 138
MvStel
Stellaria sp. TG 325
Dcarthusianorum TG 66
1.00/87/83
Dcarthusianorum 7515
Dsylvestris 6740
Dcarthusianorum 7022
Dcarthusianorum 7042
MvDsp1
Dsylvestris 6733
Dsylvestris 9111
Dsylvestris 9119
Dsylvestris Ds1
Dsylvestris TG 317
Dgratianopolitanus 330
1.00/81/80
Dgratianopolitanus 332
Dgratianopolitanus DgA
Dmonspessulanus 12919
Dmonspessulanus 12920
Dpyrenaicus 337
Dsuperbus 8718
MvDsp2
Dsylvestris TG 111
Dmonspessulanus TG 306
Dmonspessulanus TG 328
Grepens Gr137
Grepens Gr6
Grepens M21
Dcarthusianorum 30901
MvDc
Dcarthusianorum 30902
1.00/87/85
Dcarthusianorum 31602
Dcarthusianorum TG 63
Dcarthusianorum TG 65
0.1
