# Supplementary material for: Cophylogeny of the anther smut fungi and their caryophyllaceous hosts: Prevalence of host shifts and importance of delimiting parasite species for inferring cospeciation
Source: BMC Evol Biol. 2008 Mar 27;8:100. doi: 10.1186/1471-2148-8-100 (PMC2324105; doi:10.1186/1471-2148-8-100)
Supplement: Additional file 4 — Host species, name and sampling localities of the Microbotryum smut fungi analysed in this study. [file 1471-2148-8-100-S4.doc]

### Additional file 4 – Host species, name and sampling localities of the *Microbotryum* smut fungi analysed in this study.

| Host species | Name | Sampling localities | Strains sequenced in a previous study [29] |
| --- | --- | --- | --- |
| *D. carthusianorum* | 7022 | Flafleralp, The Alps, Switzerland | X |
| *D. carthusianorum* | 7042 | Flafleralp, The Alps, Switzerland | X |
| *D. carthusianorum* | 7515 | Blatten, The Alps, Switzerland | X |
| *D. carthusianorum* | 30901 | Val d'Esquierry, The Pyrénées, France | X |
| *D. carthusianorum* | 30902 | Val d'Esquierry, The Pyrénées, France | X |
| *D. carthusianorum* | 31602 | Grosio, The Alps, Italy | X |
| *D. carthusianorum* | TG-63 | Leukerbad, The Alps, Switzerland |  |
| *D. carthusianorum* | TG- 65 | Leukerbad, The Alps, Switzerland |  |
| *D. carthusianorum* | TG- 66 | Albinen, The Alps, Switzerland |  |
| *D. carthusianorum* | TG- 329 | Rheinland, Altenahr, Germany |  |
| *D. gratianopolitanus* | 330 | Puy de Sancy, Massif Central, France |  |
| *D. gratianopolitanus* | TG- 331 | Yverdon, Jura, Switzerland |  |
| *D. gratianopolitanus* | 332 | Yverdon, Jura, Switzerland |  |
| *D. gratianopolitanus* | Dga | Le Chasseron, Jura, Switzerland | X |
| *D. monspessulanus* | 12919 | Gavarnie, The Pyrénées, France | X |
| *D. monspessulanus* | 12920 | Gavarnie, The Pyrénées, France | X |
| *D. monspessulanus* | TG -305 | Route d'orédon, The Pyrénées, France |  |
| *D. monspessulanus* | TG-306 | Val d'Esquierry, The Pyrénées, France |  |
| *D. monspessulanus* | TG- 328 | Puy Mary, Massif Central, France |  |
| *D. superbus* | 8718 | Davos, The Alps, Switzerland | X |
| *D. sylvestris* | 6733 | Leuk-Gambel, The Alps, Switzerland | X |
| *D. sylvestris* | 6740 | Leuk-Gambel, The Alps, Switzerland | X |
| *D. sylvestris* | 9111 | Zernez, The Alps, Switzerland | X |
| *D. sylvestris* | 9119 | Zernez, The Alps, Switzerland | X |
| *D. sylvestris* | Ds1 | The Alps, France | X |
| *D. sylvestris* | *Dsylvestris*_us1 | The Alps, France |  |
| *D. sylvestris* | TG-111 | Lozère, Massif Central, France |  |
| *D. sylvestris* | TG-317 | Grosio, The Alps, Italy |  |
| *D. sylvestris* | TG-331 | Yverdon, Jura, Switzerland |  |
| *G. repens* | M21 | Grosio, The Alps, Italy |  |
| *G. repens* | Gr6 | Grosio, The Alps, Italy | X |
| *G. repens* | Gr137 | Grosio, The Alps, Italy | X |
| *L. flos-cuculi* | 9203 | Tschierv, The Alps, France | X |
| *L. flos-cuculi* | 9204 | Tschierv, The Alps, France |  |
| *L. flos-cuculi* | 9205 | Tschierv, The Alps, France | X |
| *L. flos-cuculi* | 10916 | Einsiedeln, Zurich, Switzerland |  |
| *L. flos-cuculi* | 10934 | Gäbrisseeli, The Alps, Switzerland | X |
| *L. flos-jovis* | M26 | Guarda, The Alps, Switzerland |  |
| *L. flos-jovis* | M27 | Guarda, The Alps, Switzerland |  |
| *L. flos-jovis* | 410 | Guarda, The Alps, Switzerland |  |
| *S. acaulis* | 31401 | Cirque de Troumouse, The Pyrénées, France | X |
| *S. acaulis* | 31402 | Cirque de Troumouse, The Pyrénées, France | X |
| *S. acaulis* | Sa1 | Oberalppass, The Alps, Switzerland | X |
| *S. acaulis* | Sa2 | Oberalppass, The Alps, Switzerland | X |
| *S. acaulis* | 89_01 | Flüelapass, The Alps, Switzerland |  |
| *S. acaulis* | 89_01 | Flüelapass, The Alps, Switzerland |  |
| *S. acaulis* | 339 | Berninapass, The Alps, Switzerland |  |
| *S. acaulis* | 380 | Colorado, USA |  |
| *S. caroliniana* | Sc1 | Virginia, USA | X |
| *S. caroliniana* | Sc2 | Virginia, USA | X |
| *S. caroliniana* | TG-117sc | Virginia, USA | X |
| *S. dioica* | 7212 | Flafleralp, The Alps, Switzerland | X |
| *S. dioica* | 7237 | Flafleralp, The Alps, Switzerland | X |
| *S. dioica* | Sdb | Taulé, Morlaix, Brittany, France | X |
| *S. latifolia* | 4001 | Auffargis, Essonne, France | X |
| *S. latifolia* | 4106 | Senlisse, Essonne, France | X |
| *S. latifolia* | 10002 | Tirano, The Alps, Italy | X |
| *S. latifolia* | 10006 | Tirano, The Alps, Italy | X |
| *S. latifolia* | Sl1 | Lamole, Italy | X |
| *S. latifolia* | Sl2 | Mountain Lake, Virginia, USA | X |
| *S. lemmonii* | Sle1 | Virginia, USA | X |
| *S. nutans* | 7901 | Andermatt, The Alps, Switzerland | X |
| *S. nutans* | 8742 | Davos, The Alps, Switzerland | X |
| *S. nutans* | Lb | Jura, France | X |
| *S. nutans* | TG 303 | Lac d'Orédon, The Pyrénées, France |  |
| *Sa. ocymoïdes* | Soc1 | Guarda, The Alps, France | X |
| *Sa. ocymoïdes* | 96_02 | Tirano, The Alps, Italy |  |
| *Sa. officinalis* | R2 | Tirano, The Alps, Italy | X |
| *Sa. officinalis* | TG-101 | Orsay, Essonne, France |  |
| *Sa. officinalis* | TG-112 | St Cirq Lapopie, Lot, France |  |
| *Sa. officinalis* | TG-116 | Virginia USA |  |
| *Sa. officinalis* | TG-124 | Sazos, The Pyrénées, France |  |
| *Sa. officinalis* | TG-135 | Sazos, The Pyrénées, France |  |
| *Sa. officinalis* | TG-138 | Basse Terre, Isère, France |  |
| *Sa. officinalis* | TG-341 | Bourgogne, Rémigny, France |  |
| *Sa. officinalis* | TG-343 | Orsay, Essonne, France |  |
| *S. otites* | M19 | Grosio, The Alps, Italy |  |
| *S. otites* | 338 | Yverdon, Jura, Switzerland |  |
| *S. otites* | Sott | The Alps, France | X |
| *A. rupestris*  *(S. rupestris)* | 383 | The Alps, France |  |
| *A. rupestris*  *(S. rupestris)* | 105-02 | Machanebal, The Alps, Switzerland |  |
| *S. virginica* | Svi1 | Virginia, USA | X |
| *S. virginica* | TG-117sv | Virginia, USA |  |
| *S. virginica* | 387 | C'ville Res, USA |  |
| *S. vulgaris* | 7806 | Andermatt, The Alps, Switzerland | X |
| *S. vulgaris* | 7807 | Andermatt, The Alps, Switzerland | X |
| *S. vulgaris* | 7903 | Andermatt, The Alps, Switzerland | X |
| *S. vulgaris* | 7913 | Andermatt, The Alps, Switzerland | X |
| *S. vulgaris* | 30027 | Pic du midi de Bigorre, The Pyrénées, France | X |
| *S. vulgaris* | 30030 | Pic du midi de Bigorre, The Pyrénées, France | X |
| *Stellaria* sp. | TG-325 | Brønderslev, Jutland, Denmark |  |

Strains that had been previously analysed in [29] are indicated. Strains are stored in the ESE lab in Orsay and are available upon request.
