# Supplementary material for: Cophylogeny of the anther smut fungi and their caryophyllaceous hosts: Prevalence of host shifts and importance of delimiting parasite species for inferring cospeciation
Source: BMC Evol Biol. 2008 Mar 27;8:100. doi: 10.1186/1471-2148-8-100 (PMC2324105; doi:10.1186/1471-2148-8-100)
Supplement: Additional file 5 — Sampling localities of plant samples analysed in this study. [file 1471-2148-8-100-S5.doc]

### Additional file 5 – Sampling localities of plant samples analysed in this study.

| Plant species | Sampling localities |
| --- | --- |
| *Dianthus carthusianorum* | Leuk, Valais, Switzerland |
| *D. gratianopolitanus* | Yverdon, Switzerland |
| *D. monspessulanus* | Monte Baldo, Veneto, Italy |
| *D. superbus* | St. Moritz, Grisons, Switzerland |
| *D. sylvestris* | Guarda, Grisons, Switzerland |
| *Gypsophila repens* | Grosio, Valtellina, Italy |
| *Lychnis flos-cuculi* | Grüningen, Zürich, Switzerland |
| *L. flos-jovis* | Guarda, Grisons, Switzerland |
| *Saponaria ocymoides* | Guarda, Grisons, Switzerland |
| *Sa. officinalis* | Eggerberg, Valais, Switzerland |
| *Silene acaulis* | Maloja, Grisons, Switzerland |
| *S. caroliniana* | California, USA |
| *S. dioica* | Simplonpass, Valais, Switzerland |
| *S. latifolia* | Brentjong, Valais, Switzerland |
| *S. lemmonii* | Virginia, USA |
| *S. nutans* | Brig, Valais, Switzerland |
| *S. otites* | Grosio, Valtellina, Italy |
| *Atocion rupestris (=S. rupestris)* | Berninapass, Grisons, Switzerland |
| *S. virginica* | Virginia, USA |
| *S. vulgaris* | Davos, Grisons, Italy |
| *Stellaria* sp. | Brønderslev, Jutland, Denmark |

Dry leafs are stored in the ESE lab in Orsay and are available upon request.
